# Contact electrification induced interfacial reactions and direct electrochemical nanoimprint lithography in n-type gallium arsenate wafer†
†Electronic supplementary information (ESI) available: Electrochemical measurements of the interfaces, optimization of the contact force and temperature of ECNL, XPS analysis, and more examples of ECNL on n-GaAs. See DOI: 10.1039/c6sc04091h



**DOI:** 10.1039/c6sc04091h

**Published:** 2016-12-16

**Authors:** Jie Zhang, Lin Zhang, Wei Wang, Lianhuan Han, Jing-Chun Jia, Zhao-Wu Tian, Zhong-Qun Tian, Dongping Zhan

**Affiliations:** a State Key Laboratory of Physical Chemistry of Solid Surfaces (PCOSS) , Collaborative Innovation Centre of Chemistry for Energy Materials (iChEM) , Department of Chemistry , College of Chemistry and Chemical Engineering , Xiamen University , Xiamen 361005 , China . Email: dpzhan@xmu.edu.cn

## Abstract

Although metal assisted chemical etching (MacEtch) has emerged as a versatile micro-nanofabrication method for semiconductors, the chemical mechanism remains ambiguous in terms of both thermodynamics and kinetics. Here we demonstrate an innovative phenomenon, *i.e.*, the contact electrification between platinum (Pt) and an n-type gallium arsenide (100) wafer (n-GaAs) can induce interfacial redox reactions. Because of their different work functions, when the Pt electrode comes into contact with n-GaAs, electrons will move from n-GaAs to Pt and form a contact electric field at the Pt/n-GaAs junction until their electron Fermi levels (*E*_F_) become equal. In the presence of an electrolyte, the potential of the Pt/electrolyte interface will shift due to the contact electricity and induce the spontaneous reduction of MnO_4_^–^ anions on the Pt surface. Because the equilibrium of contact electrification is disturbed, electrons will transfer from n-GaAs to Pt through the tunneling effect. Thus, the accumulated positive holes at the n-GaAs/electrolyte interface make n-GaAs dissolve anodically along the Pt/n-GaAs/electrolyte 3-phase interface. Based on this principle, we developed a direct electrochemical nanoimprint lithography method applicable to crystalline semiconductors.

## Abstract 2

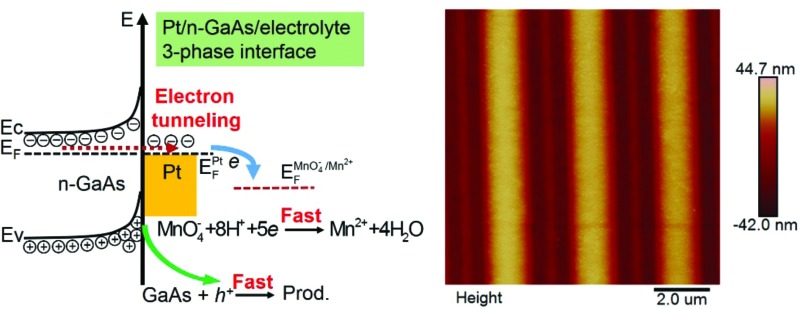
We demonstrated contact electrification induced interfacial redox reactions and developed a direct electrochemical nanoimprint lithography method applicable to crystalline semiconductors.

## Introduction

**Fig. 1:**
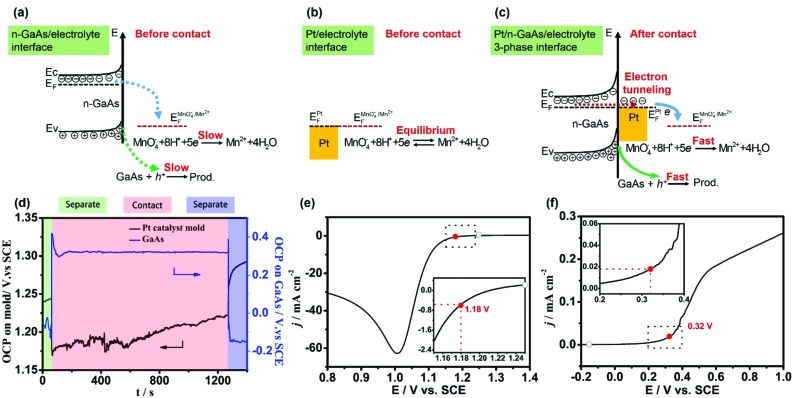
(a) The diagram of energy levels at the n-GaAs/electrolyte interface. (b) The diagram of energy levels at the Pt/electrolyte interface. (c) The diagram of energy levels at the Pt/n-GaAs/electrolyte 3-phase interface. (d) The interfacial potentials measured at the separate–contact–separate status in an aqueous solution containing 1.84 mol L^–1^ H_2_SO_4_ and 0.040 mol L^–1^ KMnO_4_ with a Pt mold electrode (area: 1 cm^2^). (e and f) The linear-scan voltammograms obtained with a Pt disk electrode (diameter: 2 mm) and a n-GaAs electrode (area: 0.6 cm^2^) in the same solution with a scan rate of 100 mV s^–1^. Insets are the magnification of the segments marked by the dotted rectangles.

Mass production of three dimensional micro-nanostructures (3D-MNS) is demanded in industrial domains, such as ultra large scale integration circuit (ULSI),^1^ microelectromechanical systems (MEMS),^2^ miniaturized total analysis systems (μ-TAS)^3^ and precision optics.^4^ Metal assisted chemical etching (MacEtch) has emerged as a wet chemical etching method for the 3D-MNS fabrication on semiconductor materials with nanoscale resolution, since its first report in 2000.^5–8^ Metal nanoparticles or nanopatterns (*e.g.*, Pt, Au, Ag, Pd, *etc.*) are deposited on the semiconductor surface by self-assembly or photolithography. With strong oxidants such as KMnO_4_, NaS_2_O_4_, K_2_CrO_7_ or H_2_O_2_, porous or erected nanostructures can be obtained.^9–18^ Although it has been extensively discussed, the chemical mechanism of MacEtch still remains ambiguous. It is well accepted that metals can catalyze the oxidative half-reaction by injecting positive holes into semiconductors.^10,19–22^ Recently, a Schottky barrier catalysis mechanism was proposed to elucidate the spatial distribution of the injected holes.^23^ In terms of thermodynamics, the semiconductors can be oxidized possibly by the oxidants because the reduction potential of the oxidants are much higher than those of the anodic dissolution of the semiconductors. However, this hardly happens in the case of the kinetics due to the slow reaction rate. In fact, neither the cathodic reduction of oxidants nor the anodic dissolution of semiconductors would happen at the isolated metal/electrolyte interface and the isolated semiconductor/electrolyte interface (Fig. 1a and b). Why can the half-reactions happen spontaneously at the 3-phase metal/semiconductor/electrolyte interface? By which pathway are the electrons transported or the positive holes injected? What is the rate determining step in the MacEtch processes?

To answer these questions, we adopt a metal electrode or metalized imprint mold separated from the semiconductor substrate to construct the metal/semiconductor/electrolyte 3-phase interface. In the conventional MacEtch configuration, metal catalysts are deposited directly on the semiconductor surface.^24–26^ Although it is well accepted that interfacial charge transfers are involved in MacEtch, it is difficult to obtain the direct electrochemical information. Most mechanisms are based on analysis of the thermodynamics. Here, in the separated configuration, the direct electrochemical measurements can be performed not only at the isolated semiconductor/electrolyte and the isolated metal/electrolyte interfaces, but also at the 3-phase interface of MacEtch system. We demonstrate that the contact electrification between the semiconductor and the metal plays an important role in MacEtch. Because of the contact electric field, the potential of the metal/electrolyte interface shifts cathodically and induces the reduction reaction of the oxidant, and the semiconductor/electrolyte interface is polarized anodically, resulting in the oxidative dissolution of the semiconductor.

Based on this configuration, we developed a direct electrochemical nanoimprint lithography (ECNL) method applicable to crystalline semiconductors. Nanoimprint lithography (NIL) was proposed in 1995 and plays important roles in the fabrication of 3D-MNS due to its high throughput, high resolution and low cost.^27–30^ As a mechanical pressing method, NIL applies only to thermoplastic and photocuring resists.^31,32^ Direct NIL on a semiconductor substrate was first proposed as a laser-assisted direct imprint (LADI) method in 2002.^33^ The key issues of LADI include the injection of a pulsed laser beam extremely powerfully to “melt” the Si substrate and the high pressure (>1 MPa) used to press the 3D-MNSs. The second case using a MacEtch configuration was reported very recently.^34^ Unfortunately, the author neglected the chemical kinetics of MacEtch and claimed that only porous silicon was applicable due to the mass balance problem. From the viewpoint of electrochemistry, we demonstrate that ECNL can be applied to crystalline semiconductors as a direct nanoimprint lithography technique.

## Results and discussion

When two objects come into contact with each other, they should have equal Fermi levels or, strictly, electrochemical potentials. Electrons will transfer from the object with a lower work function to the other until their Fermi levels become equal. Thus, a contact electric field and a contact potential will be formed at the interface. This phenomenon is historically called “contact electrification”. Recently, the contact electrification of bimetallic nanoparticles, including contact potential, surface charging and Fermi level equilibration, have been well discussed and found to be valuable in nanocatalysis as well as in nanophotonics.^35–39^ Here we will discuss the interfacial redox reactions induced by the contact electrification between a metal and semiconductor.

Fig. 1a shows the energy levels at the n-GaAs/electrolyte interface. Since the measured open circuit potential (OCP: –0.15 V, Fig. 1d) doesn't match the reaction potentials of both MnO_4_^–^ and GaAs (Fig. 1e and f), the direct oxidation of n-GaAs by MnO_4_^–^ is slow kinetically. Fig. 1b shows the energy levels at the Pt/electrolyte interface. Since Pt is a good catalyst for the reduction of MnO_4_^–^, the measured OCP (1.24 V, Fig. 1d) is actually the formal potential of MnO_4_^–^ at thermodynamic equilibrium. There is no net reduction of MnO_4_^–^ once the equilibrium is achieved. From the linear-scan voltammograms obtained at the isolated interfaces (the hollow circle symbols in Fig. 1e and f), neither the oxidation of GaAs nor the reduction of MnO_4_^–^ would happen at the isolated n-GaAs/electrolyte interface and Pt/electrolyte interface, respectively.

However, when the n-GaAs substrate comes into compact contact with the Pt mold electrode (Fig. 1c), electrons will transfer from n-GaAs to Pt because the electron work function of n-GaAs is lower than that of Pt.^40–43^ Thus, a contact electric field will be formed at the Pt/n-GaAs junctions and the contact potential is determined by the difference in work functions of Pt and n-GaAs. At equilibrium, the electron Fermi level (*E*_F_) of n-GaAs will be equal to that of Pt.^41^ Since n-GaAs is highly doped by silicon ((0.8–2.3) × 10^18^ cm^–3^), the ohmic contact provides an electron tunneling pathway across the Pt/n-GaAs interface. If the aligned *E*_F_ matches that of the MnO_4_^–^ anions in the electrolyte, MnO_4_^–^ anions will be reduced at the Pt/electrolyte interface, and electrons in n-GaAs will flow into Pt and be taken away by MnO_4_^–^ anions (Fig. 1c). As a result, the accumulated positive holes in n-GaAs will make the local area dissolve along the Pt/n-GaAs/electrolyte 3-phase interface.

The theoretical analysis is well verified by the experimental results obtained at 35–37 °C in an aqueous solution containing 1.84 mol L^–1^ H_2_SO_4_ and 0.040 mol L^–1^ KMnO_4_. When the Pt mold electrode (area: 1 cm^2^) comes into contact with the n-GaAs substrate through a SECM positioning system, both potentials change dramatically. At a constant pressure of 0.5 atm, the potential of the Pt/electrolyte interface steps from +1.24 V to +1.18 V, and the potential of the n-GaAs/electrolyte interface steps from –0.15 V to +0.41 V and then is stabilized at +0.32 V (Fig. 1d). The potential shifts are crucial because MnO_4_^–^ anions can be reduced at +1.18 V on the Pt electrode and n-GaAs can dissolve anodically at +0.32 V (the red symbols in Fig. 1e and f). When the Pt mold electrode and the n-GaAs are separated, the interfacial potentials will move to where the half-reactions are both halted (Fig. 1d).

From the experimental results shown in Fig. 1, it can be concluded that the contact electrification between the Pt and n-GaAs plays an important role in the occurrence of the following two half-reactions:

1 MnO_4_^–^ + 8H^+^ + 5e → Mn^2+^ + 4H_2_O

2 GaAs + 3H_2_O → Ga^3+^ + AsO_3_^3–^ + 6H^+^ + 6e

On comparing the insets in Fig. 1e and f, the marked current densities are found to be mismatched, showing that the reaction rate of the Pt catalysed reduction of MnO_4_^–^ at 1.18 V is much faster than that of the GaAs anodic dissolution at 0.32 V. However, when the Pt mold electrode comes into contact with the n-GaAs substrate, the contact electrification induced MnO_4_^–^ reduction and n-GaAs oxidation should have the same reaction rate according to the charge neutrality principle. The reduction rate of MnO_4_^–^ should be slowed down due to the slow kinetics of GaAs oxidation. This means that a smaller overpotential is needed for MnO_4_^–^ reduction during the MacEtch process. This might be the reason why the measured potential of the Pt/electrolyte interface keeps shifting positively. The potential oscillation may imply a non-equilibrium corrosion processes worthy of future research.

**Fig. 2:**
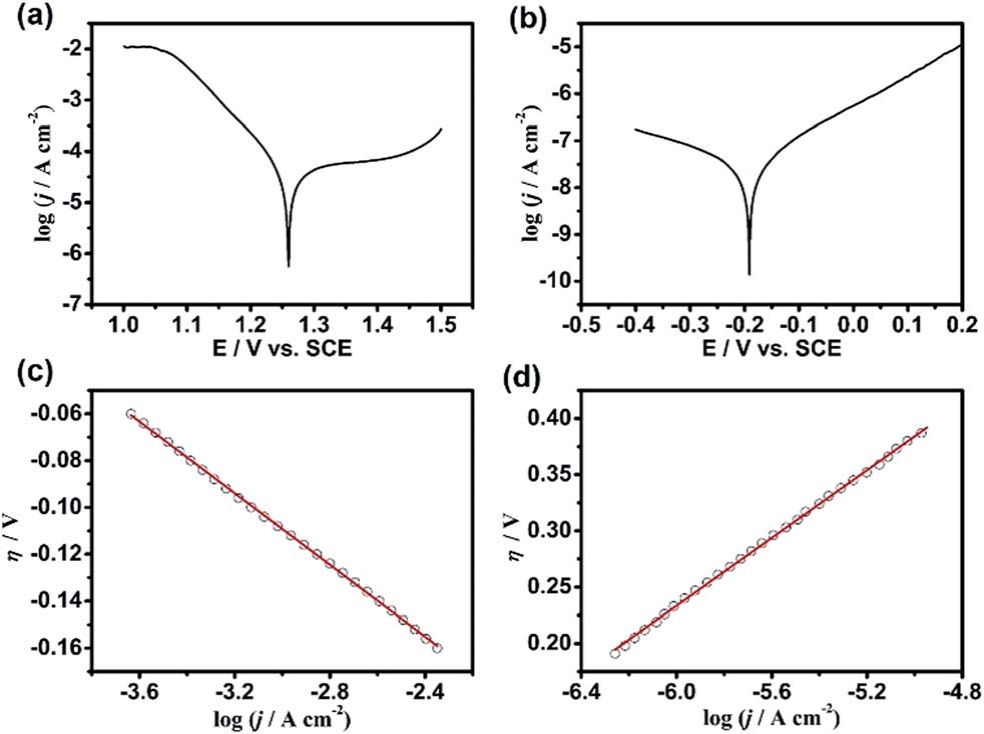
(a and b) The Tafel curves of a 2 mm-diameter Pt electrode and n-GaAs electrode with area of 0.6 cm^2^ in the working solution containing 0.040 mol L^–1^ KMnO_4_ and 1.84 M H_2_SO_4_ at 35–37 °C. (c and d) The semi-logarithmic relationship between the overpotential and the current for the Pt electrode and n-GaAs electrode.

To elucidate the kinetic properties of this MacEtch system, Tafel experiments were performed in the same solution with a Pt disc electrode (diameter: 2 mm) and a n-GaAs electrode (area: 0.6 cm^2^), respectively. Although both half-reactions are irreversible, the cathodic polarization curve of MnO_4_^–^ reduction and the anodic polarization curve of n-GaAs dissolution should obey Tafel's law.^44,45^ The exchange current density (*j*_0_) of MnO_4_^–^ reduction at the Pt/electrolyte interface can be obtained as 3.74 × 10^–5^ A cm^–2^ (Fig. 2a and c) as well as the *j*_0_ of n-GaAs dissolution at n-GaAs/electrolyte interface as 2.82 × 10^–8^ A cm^–2^ (Fig. 2b and d). Comparing the *j*_0_ values, the rate determining step (rds) is proved to be the anodic dissolution of n-GaAs. More discussion on the Tafel experiments can be seen in Fig. S1.†

**Fig. 3:**
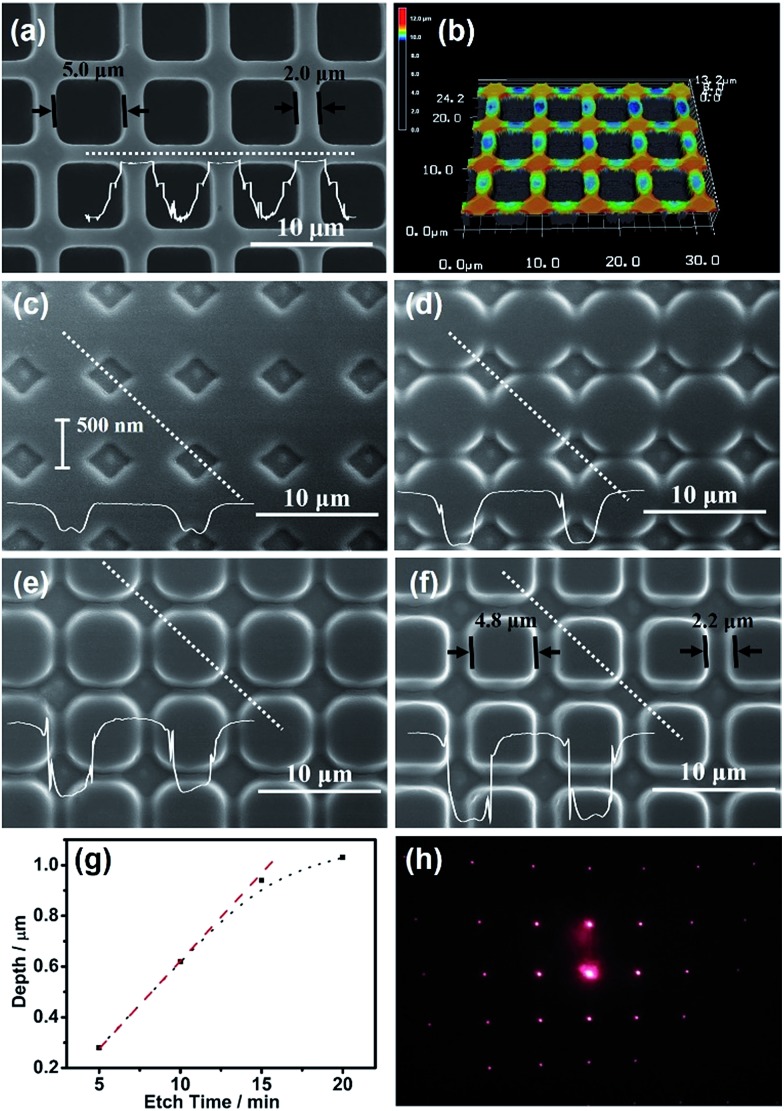
(a) The SEM image of the Pt metalized PMMA mold. (b) The confocal laser microscopy image (*i.e.*, 3D height image) of (a). (c–f) The imprinted microstructures on n-GaAs obtained in 5, 10, 15 and 20 minutes. (g) The relationship between the vertical etch depth and etching time. (h) The diffraction pattern of the microstructures in (f) illuminated vertically by a 650 nm laser beam, the light intensity is 5 mW. The insets are the cross-sectional profiles of the locations marked by dotted lines.

The contact electrification inducing the interfacial redox reactions has very good spatial resolution, *i.e.*, the anodic dissolution of n-GaAs occurs only along the Pt/n-GaAs/electrolyte 3-phase interface. A Pt mold electrode (area: 1 cm^2^) with multistage 3D-MNS was adopted as the imprint mold, where the intersections were the highest parts (Fig. 3a and b). When the imprint mold comes into contact with the n-GaAs, with an optimized pressure (0.5 atm), the anodic dissolution of n-GaAs began at the highest parts because the intersections were imprinted into the n-GaAs wafer in the first 5 min (Fig. 3c). With the time going on, square frames are imprinted gradually into the n-GaAs wafer in 20 min (Fig. 3d–f). The sizes of the squares and frames on the imprint mold are 5.0 μm and 2.0 μm (Fig. 3a). The corresponding sizes of the imprinted squares and frames in the n-GaAs wafer were measured with a value of 4.8 μm and 2.2 μm. The machining tolerance is caused by the mobility of positive holes on n-GaAs, which is predicted to be not beyond the Debye length.^46^ The results prove that the anodic dissolution of n-GaAs is confined at the Pt/n-GaAs/electrolyte 3-phase interface. The optimization of technical parameters such as imprinting pressure and working temperature are described in the ESI (Fig. S2–S5†).

The “W”-shaped profile (Fig. 3c), also reported by Ferreira's group, indicates that the anodic dissolution of GaAs begins at the edge of the 3-phase interface and goes gradually into the inner region. The vertical etching rate, characterized by the etch depth as a function of etching time, is constant with a value of 66 nm min^–1^ in the first 15 min (Fig. 3g). The decreased etching rate after 15 min might be attributed to the consumption of KMnO_4_ in the ultrathin electrolyte layer between the imprint mold electrode and n-GaAs wafer as well as the passivation of the n-GaAs surface.^47^ The blocked diffusion of etching products in the ultrathin electrolyte layer between the Pt imprint mold and n-GaAs wafer might induce the precipitation of Ga_2_O_3_ and AsO_*X*_ (see XPS analysis in Fig. S6†). Nevertheless, the trace amount of oxides have little effect on the MacEtch process because of the electron tunneling effect between Pt and n-GaAs.^48^ The XPS results don't show MnO_2_ on the n-GaAs surface, indicating that MnO_4_^–^ is reduced directly to soluble Mn^2+^ on the Pt surface. Fig. 3h shows the diffraction pattern of the obtained 3D-MNS shown in Fig. 3f, indicating that the contact electrification induced interfacial redox reactions have potential applications in the fabrication of optic microdevices on functional semiconductors with the working mode of nanoimprint lithography.

**Fig. 4:**
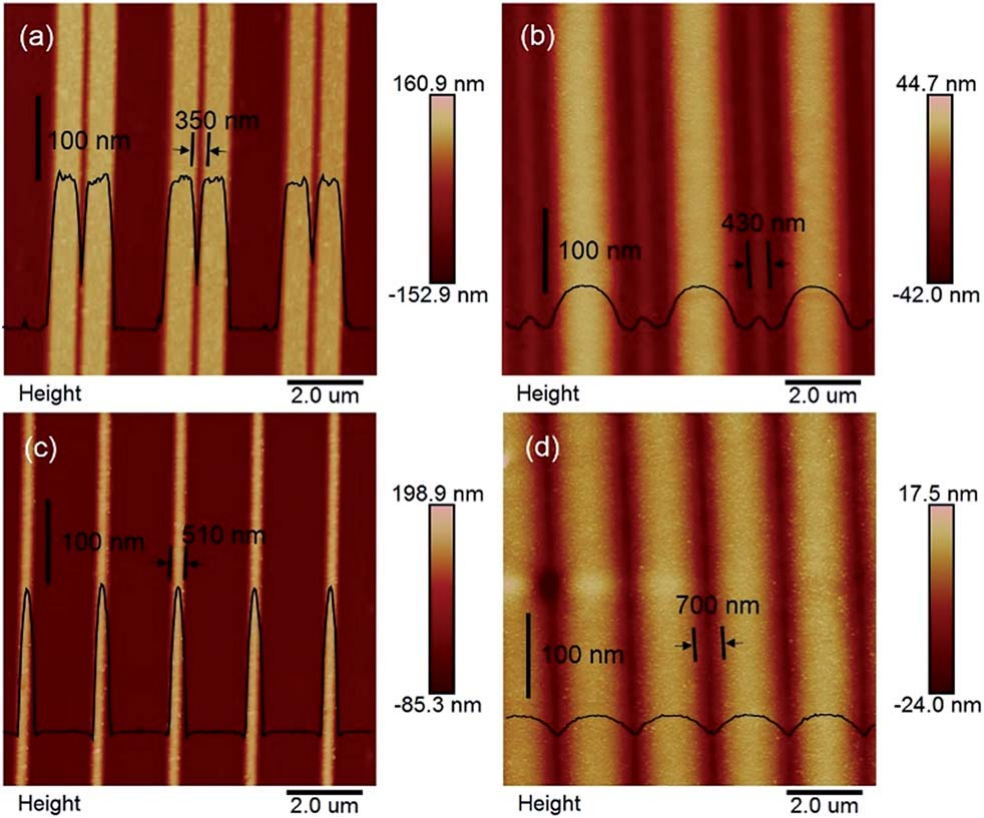
(a) The AFM image of nano-grooves on the mold. (b) The AFM image of nano-grooves transferred onto n-GaAs. (c) The AFM image of nanowires on the mold. (d) The AFM image of nanowires transferred onto n-GaAs.

At the Pt/GaAs/electrolyte 3-phase interface, the interfacial redox reactions are totally spontaneous without any external energy. Based on this unique principle, NIL can be performed directly on the n-GaAs wafer without involving a laser, heat, light or high pressure, not to mention photocuring resists and thermoplastic resists. We define this technique as ECNL, through which 3D-MNS can be fabricated on a n-GaAs wafer as shown in Fig. 4. The 350 nm-width nano-grooves (Fig. 4a) were imprinted into a n-GaAs wafer with a width of 430 nm (Fig. 4b). 510 nm-width nanowires (Fig. 4c) were imprinted into a n-GaAs wafer with a width of 700 nm (Fig. 4d). The AFM profiles show that the obtained nanostructures present continuously curved surfaces, and the etch depth is as small as 53 nm (Fig. 4b) and 22 nm (Fig. 4d). The results show that the ECNL technique is good at fabricating bas-relief 3D-MNSs. In order to fabricate high aspect ratio 3D-MNSs, the mass transfer problem, including both the supply of oxidant (MnO_4_^–^) and the removal of the products (Ga^3+^ and AsO_3_^3–^), should be solved in future through the optimization of the imprinted mold as well as the introduction of micro-nanofluidic techniques. For more ECNL examples, please see Fig. S7 and S8.†

## Conclusions

In conclusion, we propose that the chemical mechanism of MacEtch is contact electrification induced interfacial redox reactions. When the metal comes into contact with the semiconductor, at equilibrium they will achieve the same electron Fermi level, resulting in contact electrification of the metal/semiconductor interface. In the case of highly doped semiconductors, the ohmic contact provides an electron tunneling pathway at the metal/semiconductor interface. The contact electrification between the metal and semiconductor makes the potential of the metal/electrolyte interface shift and start the cathodic reduction of the oxidants in the electrolyte. The electrons in the n-type semiconductor are transported to the metal and then transferred to the oxidant. Meanwhile, the accumulated positive holes induce the anodic dissolution of the semiconductor along the metal/semiconductor/electrolyte 3-phase interface. The MacEtch process will keep going because the 3-phase interface is always renewed due to the anodic dissolution of the semiconductor. Based on this principle, we developed a direct electrochemical nanoimprint lithography (ECNL) technique applicable to semiconductors, which has potential applications in the mass production of functional 3D-MNS with low cost and high accuracy in the semiconductor manufacturing industry.

## Experimental

### Chemicals and materials

KMnO_4_ and H_2_SO_4_ were analytical grade and provided by Sinopharm Co., China. Silicon doped n-type GaAs (100) wafers with a doping level between (0.8–2.3) × 10^18^ cm^–3^ were purchased from China Crystal Technologies Co., China. Before the experiments, n-GaAs was rinsed with acetone and deionized water. All aqueous solutions were prepared with deionized water (18.2 MΩ cm, Milli-Q, Millipore Co.).

### Instruments and procedures

All the electrochemical experiments were performed with an electrochemical workstation (CHI920c, CHI Co. USA). With the positioning system of CHI920c, the potentials of the Pt/electrolyte interface and n-GaAs/electrolyte interface were measured in both isolated and contact status. All the PMMA molds used in the experiments were prepared by nanoimprint lithography, and were then coated with titanium (thickness: 10 nm) and platinum (thickness: 100 nm) by the magnetron sputtering method. The ECNL experiments were performed using nanoimprint equipment (Eitre-6, Obducat Technologies AB, Sweden), where a special electrolytic cell is adopted to hold the MacEtch system. A force sensor was used to monitor the contact force between the mold electrode and workpiece. During the ECNL process, the contact force was kept constant with a pressure of 0.5 atm. When the ECNL process was finished, the workpiece was rinsed with deionized water and dried under nitrogen for further characterization. For more experimental details please see the ESI, S1.†

### Characterization

Confocal laser microscopy (VK-X200, KEYENCE Co.), scanning electron microscopy (Hitachi S-4800, Hitachi High-Technologies Co.) and atomic force microscopy (Nanoscope III, Digital Instrument Co.) were employed to characterize the morphology of the imprinted 3D-MNS in the semiconductors. X-ray photoelectron spectroscopy (XPS) was carried out on an Omicron Sphera II hemispherical electron energy analyzer (Monochromatic Al Kα with 1486.6 eV operating at 15 kV and 300 W). The base pressure of the systems was 5.0 × 10^–9^ mbar.
