# Supplementary material for: Contact electrification induced interfacial reactions and direct electrochemical nanoimprint lithography in n-type gallium arsenate wafer
Source: Chem Sci. 2016 Dec 16;8(3):2407–12. doi: 10.1039/c6sc04091h (PMC5369340; doi:10.1039/c6sc04091h)
Supplement: Supplementary file 1 [file SC-008-C6SC04091H-s001.pdf]

Electronic Supplementary Material (ESI) for Chemical Science.
This journal is © The Royal Society of Chemistry 2016

Supporting Information

Contact electrification Induced Interfacial Reactions and the Electrochemical Nanoimprint Lithography Directly in n-Type Gallium Arsenate Wafer

Jie Zhang, Lin Zhang, Wei Wang, Lianhuan Han, Zhao-Wu Tian, Zhong-Qun Tian, Dongping Zhan*

State Key Laboratory of Physical Chemistry of Solid Surfaces, Collaborative Innovation Center of Chemistry for Energy Materials, and Department of Chemistry, College of Chemistry and Chemical Engineering, Xiamen University, Xiamen 361005, China

Content

1 **Experimental section**

2 **Tafel curves of n-GaAs and Pt electrode in the working solution**

3 **Optimizing the contact force of ECNL**

4 **Optimizing the temperature of ECNL**

5 **XPS analysis of the n-GaAs before and after ECNL**

6 **Large area direct NIL of n-GaAs by ECNL**

7 **More examples of ECNL on n-GaAs**

1 Experimental section

Chemicals and materials

All chemicals used in the experiments ($KMnO_4$, H_2SO_4) are analytical grade or better and provided by Sinopharm Co., China. Silicon doped (n-type) GaAs (100) wafers with doping level between (0.8-2.3) × 10^{18} cm^{-3} were purchased from China Crystal Technologies Co., China. Before experiments, n-GaAs was rinsed with acetone and deionized water. All aqueous solutions were prepared with deionized water (18.2 MΩ·cm, Milli-Q, Millipore Co.).

Instruments and procedures

To investigate the change of interfacial potentials during the isolated and contact status in ECNL processes, a Pt mold electrode (area: 1 cm^2) shown in Figure 3a was controlled to bring into contact with the n-GaAs fixed at the bottom of the electrolytic cell by a micromanipulation system (SECM workstation, CHI 920c, CHI Co. USA). The interfacial potential of both the mold electrode and GaAs in the working solution containing 0.040 mol/L $KMnO_4$, 1.84 M H_2SO_4 at constant temperature (35~37℃) was monitored by the potentiostatic of CHI 920c and another potentiostate (CHI 760d), respectively. The LSV of $KMnO_4$ at Pt electrode was studied by using a Pt disk electrode (diameter: 2 mm) inserted in the same solution. Similarly, The LSV of n-GaAs in $KMnO_4$ solution was studied by using an n-GaAs electrode (area: 0.6 cm^2) inserted in the same solution. The Tafel experiments in the same solution were performed by the use of 2-mm-diameter Pt disk electrode and 0.6-cm^2-area GaAs electrode. The LSV and Tafel experiments were performed with an electrochemical workshop (CHI 920c, CHI Co. USA).

All the PMMA mold used in the experiments were prepared by nanoimprint lithography, which were then coated with titanium (thickness: 10 nm) and platinum (thickness: 100 nm) by magnetron sputtering method. The contact status is very

important for the electrochemical nanoimprint experiments. If the Pt mold electrode doesn't contact well with the GaAs substrate, it is difficult to realize the contact electrification. The MacEtch process will never occur. Our experimental condition was optimized with the contact pressure at 0.5 atm. The alignment is also crucial for the nanoimprint experiments. The key issue is to make the pressure distribution homogeneous on the GaAs substrate. For this, a gas-pressure-driven system (Obducat Eitre-6 Nanoimprint lithography system, Obducat Technologies AB, Sweden) was employed to perform the electrochemical nanoimprint experiments. The self-adapting alignment function will let the Pt mold electrode and the GaAs substrate align symmetrically with each other even if they are a little bit deviated from the horizon level. Actually, the workbench is always aligned to the horizon level before experiments. To perform the ECNL, a special electrolytic cell is adopted to hold the workpiece and the working electrolyte. A force sensor was used to monitor the contact force between mold electrode and workpiece. During the ECNL process, the contact pressure was kept constant at 0.5 atm. When the ECNL process was finished, the workpiece was rinsed with deionized water and dried under nitrogen for the further characterization.

Characterization

Confocal laser microscope (VK-X200, KEYENCE Co.), scanning electron microscope (Hitachi S-4800, Hitachi High-Technologies Co.), atomic force microscopy (Nanoscope III, Digital Instrument Co.) were employed to characterize the morphology of the fabricated micro- and nano-structures in the semiconductors. X-ray photoelectron spectroscopy (XPS) was carried out on an Omicron Sphera II hemispherical electron energy analyzer (Monochromatic Al Kα with 1486.6 eV operating at 15 kV and 300 W). The base pressure of the systems was 5.0×10^{-9} mbar.

2 Tafel curves of n-GaAs and Pt electrode in the working solution (Figures are the same as in the text, more information is given here.)

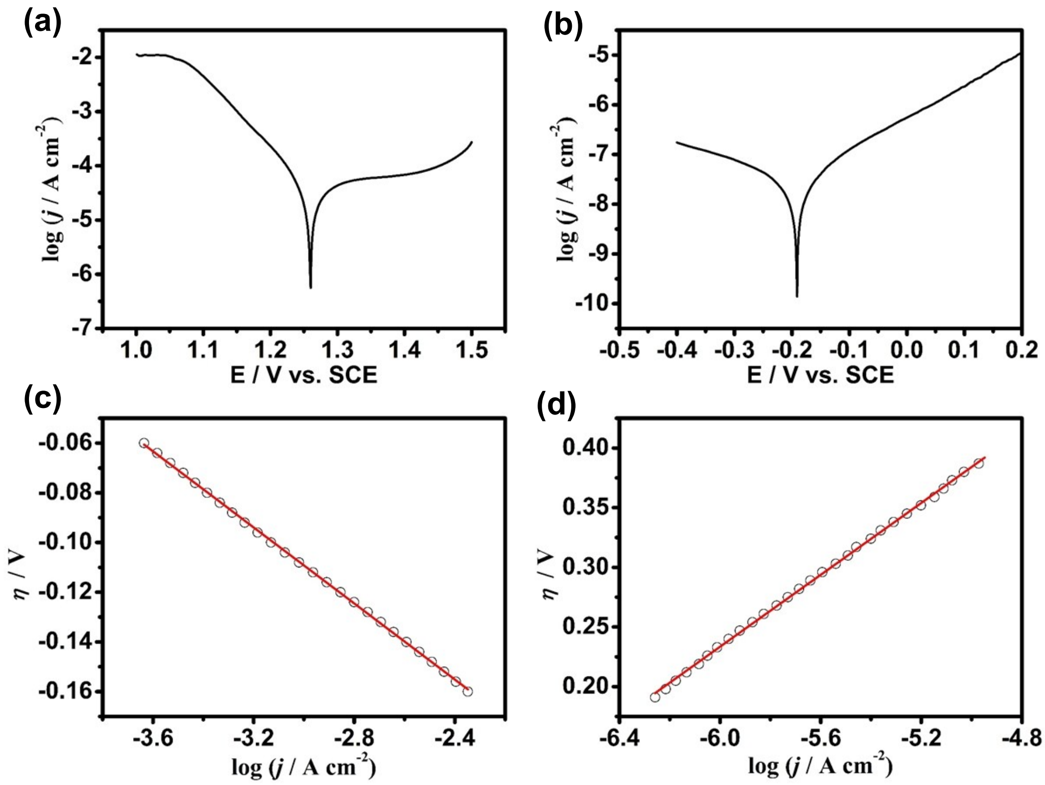

Figure S1. (a-b) the Tafel curves of n-GaAs electrode and 2-mm-diameter Pt electrode in the working solution containing 0.040 mol/L $KMnO_4$ and 1.84 mol/L H_2SO_4 at 35~37 ^{o}C. (c-d) the semi-logarithmic relationship between the potential and the current density.

Tafel experiments were performed to figure out the kinetic rate of MnO_4^- reduction at the Pt/electrotrlyte interface. From the semi-logarithmic relationship between the overpotential and the current density, exchange current density (j_0) and the product of electron transfer coefficient and electron transfer number (αn) can be obtained by Tafel equation:[1]

$$\eta = -\frac{2.303RT}{\alpha nF}\lg j + \frac{2.303RT}{\alpha nF}\lg j_0$$

For $KMnO_4$ reduction on Pt electrode, $j_0 = 3.74 \times 10^{-5}$ A/cm^2.

For the anodic dossolution of n-GaAs at the n-GaAs/electroyte interface, the anodic current density are given in a semi-logarithmic plot versus overpotential, which should abide by:[2]

$$j = j_0\left[\exp\left(\frac{nF\eta}{RT}\right)-1\right]$$

For anodic polarization ($\exp\left(\frac{nF\eta}{RT}\right) >> 1$), the equation is simplified to:

$$\eta=\frac{2.303RT}{nF}\lg j-\frac{2.303RT}{nF}\lg j_0$$

For n-GaAs anodic dissolution, j_0 = 2.82 × 10^{-8} A/cm^2. Because the electron transfer for semiconductor can occur only at the conductive band minimum or valence band maximum, the exchange current density of semiconductor electrode is much less than that of the metallic electrode.[2] Since the exchange current density of MnO_4^- reduction at the Pt/electrotrlyte interface is much higher than that of anodic dossolution of n-GaAs at the n-GaAs/electroyte interface, the rate determing step should be the anodic dissolution of GaAs.

3 Optimizing the contact pressure of ECNL

The alignment is also crucial for the nanoimprint experiments. The key issue is to make the pressure distribution homogeneous on the GaAs substrate. For the traditional MatEtch, the metal film is patterned directly on the surface of semiconductors, which ensures a good contact between the metal film and semiconductor. For the situation of ECNL herein, the contact between the mold and semiconductor is realized by the external pressure. A non-uniform contact will leads to a non-uniform etching rate, which results in a non-uniform micro-nanostructures fabricated (Figure S2).

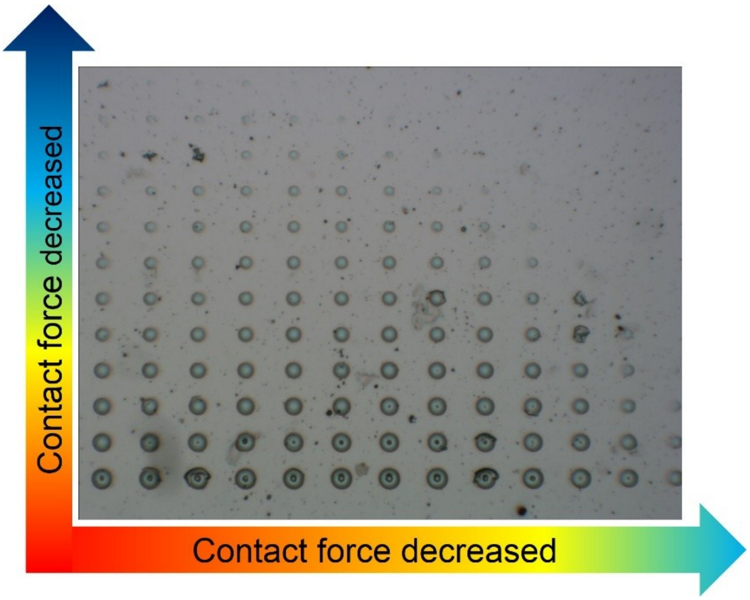

Figure S2. the non-uniform concave hemispheres fabricated under a with bad alignment. The contact pressure is 0.5 atm.

To ensure a uniform contact between mold electrode and GaAs, a gas pressure driven contact method in the nanoimprint equipment (Eitre-6, Obducat Technologies AB, Sweden) was used in the ECNL process. Before the ECNL, the mold was fixed at the bottom of the electrolytic cell, and was leveled by the bubble level. The leveling method by bubble level has some error (Figure S3). But this error has no effect on the uniformity of the contact performed by the gas pressure subsequently. The self-

adapting leveling between mold and substrate was achieved by the gas pressure driven attitude adjustment of GaAs workpiece (Figure S3). Therefore, the mold was well aligned to GaAs to ensure a uniform contact.

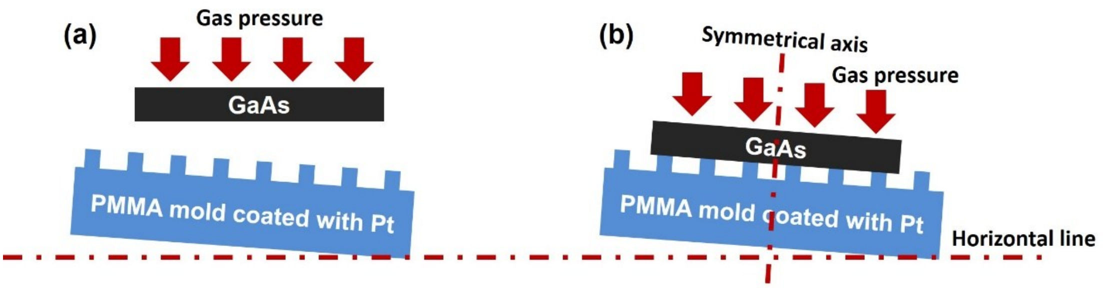

Figure S3. the schematic of self-adapting leveling between mold and substrate.

Ususally, large contact pressure (> 1 atm) will lead to the deformation and wrinkles on the imprint mold, which results in the failure of microfabrications (Figure S4). Different contact pressure are tried and the optimized contact pressure is 0.5 atm.

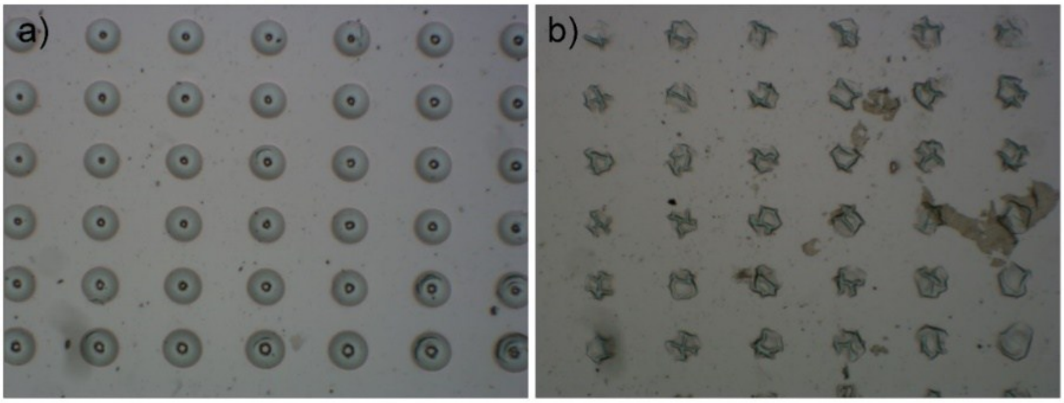

Figure S4. The optical images of concave hemispheres fabricated in n-GaAs with humps and wrinkles fabricated under a contact pressure of 1 atm (a) and 2 atm (b).

4 Optimizing the working temperature of ECNL

It should be noted that the decomposition rate of $KMnO_4$ is affected by the acidity and temperature of the working electrolyte. High concentration and high temperature should be avoided. When the temperature is higher than 40 ℃, oxygen bubbles and MnO_2 precipitatant were observed obviously in the solution containing 100 mM $KMnO_4$. To ensure the fabrication efficiency and quality, the working temperature should be kept no higher than 37 ℃ in the experiments.

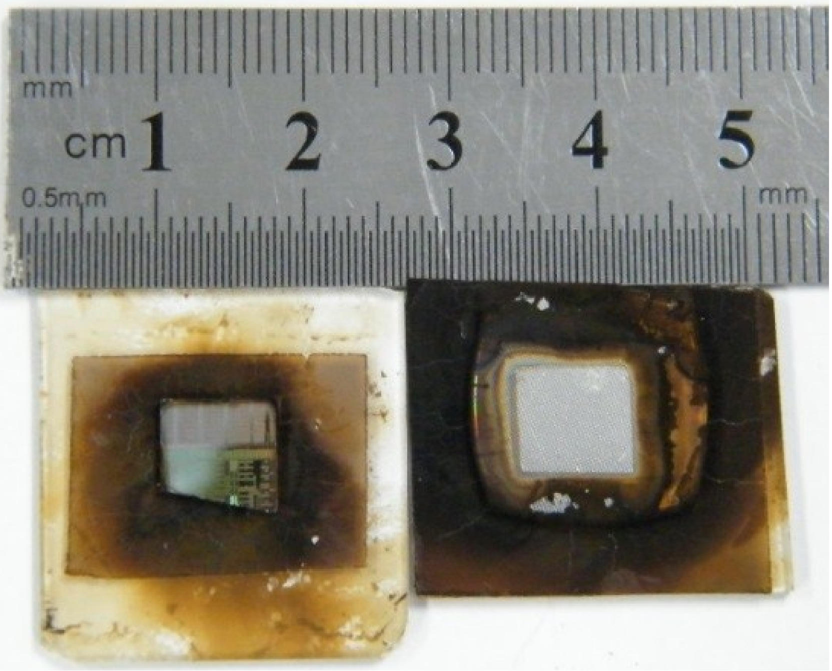

Figure S5. The precipitates of MnO_2 observed on the surface of mold after 40-min etching in the presence of 100 mM $KMnO_4$. The temperature was kept at 40 ℃.

5 XPS analysis of the n-GaAs before and after ECNL

n-GaAs can be oxidized in the air to form a little oxides on surface.[3] The bonding energy at 41.6 eV could be due to the elemental arsenic (As^0). While the bonding energy at 44.1 eV could be due to a lower arsenic oxide oxidation state (arsenic non-stoichiometric oxide, AsO_x).[4-7] After ECNL experiments, the elemental arsenic and lower arsenic oxide oxidation state increased drameticly (Figure S6d). The lower arsenic oxide oxidation state was always generated at the earlier oxidation of pure arsenic.[4, 7] The oxides As_2O_3 and As_2O_5 with bonding energy at 44.8 eV and 46.2 eV was not founed on the n-GaAs surface. Generally, arsenic oxides are more insoluble than gallium oxides.[8, 9] Therefore, the arsenic oxides were mainly formed on the surface.

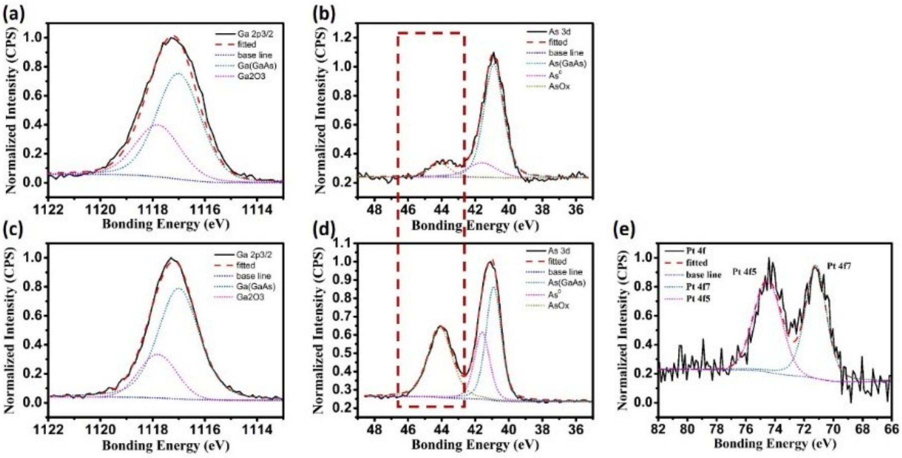

Figure S6. The X-ray photoelectron spectra of n-GaAs before (a-b) and after (c-e) the ECNL experiments. (a) and (c) XPS spectra of Ga 2p3. (b) and (d) XPS spectra of As 3d. (e) XPS spectra of Pt 4f.

For traditional MatEtch, the patterned metal on semiconductor surface will sink into the structures and should be removed by suitable etchant for the further device applications.[10] It has been well known that Pt is a better catalyst than Au and Ag in MatEtch. However, Pt is too stable to be removed than Au and Ag.[10, 11] That is why Pt is not so popular as Au. Nevertheless, in the ECNL processes, from the experimental results of the X-ray photoelectron spectroscopy (XPS) as shown in Figure S6e, little Pt is residual on the n-GaAs wafter. The average atomic concentration of platinum is almost 0.29 %. This is a distinc advantage of ECNL.

6 Large area duplication by ECNL

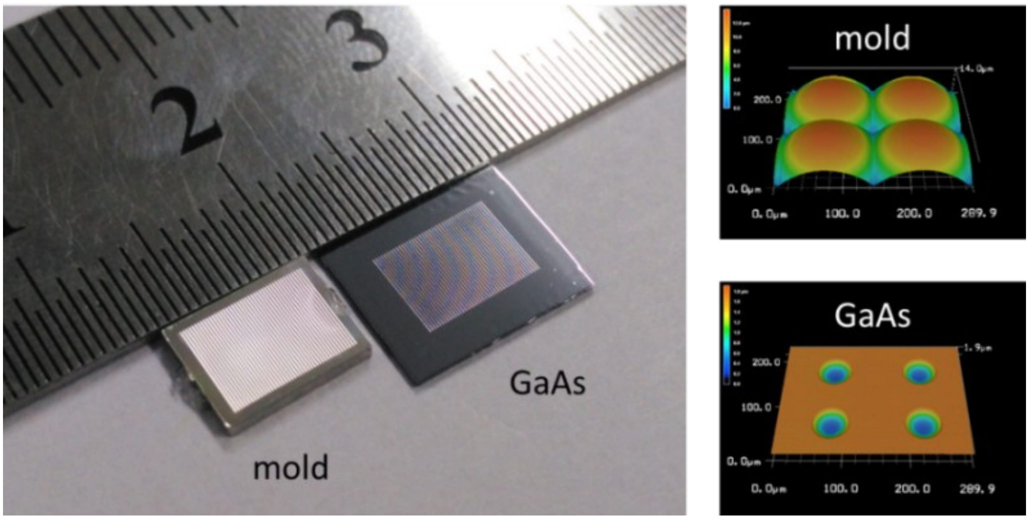

Figure S7. The left shows the large-area (~cm^2) mold with convex microlens array and the fabricated concave microlens array on n-GaAs substrate. The rights show the confocal laser microscopic images of the convex microlens on the mold and the concave microlens fabricated on the GaAs substrate.

7 More examples of ECNL on n-GaAs

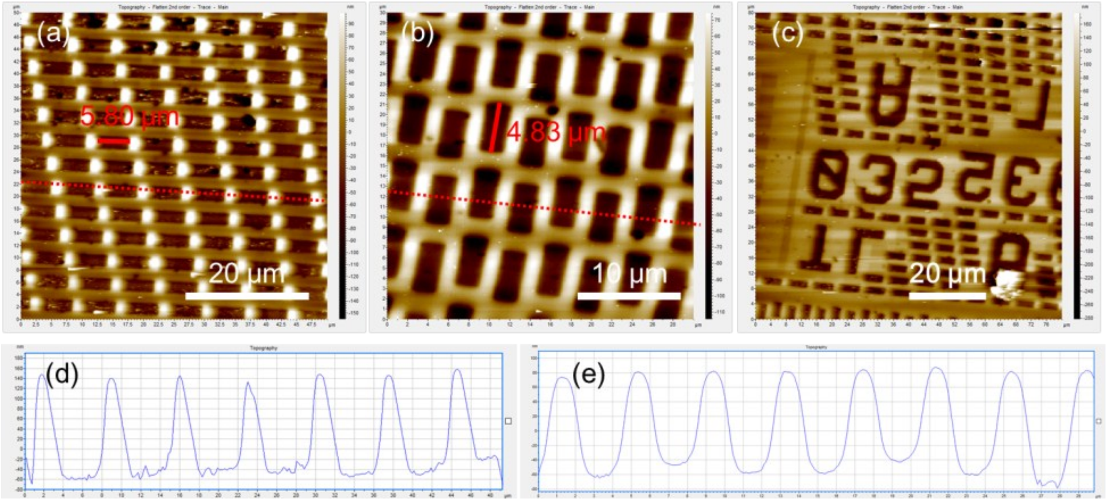

Figure S8. (a-c) Different microstructures fabricated by ECNL on n-GaAs , (d) and (e) the profiles corresponding to the red lines in (a) and (b).

Reference

1. A. J. Bard and L. R. Faulkner, *Electrochemical Methods: Fundamentals and Applications*, Wiley, New York, 2 edn., 2001.
2. R. Memming and D. Bahnemann, *Semiconductor electrochemistry*, John Wiley & Sons, 2015.
3. J. T. Wolan, C. K. Mount and G. B. Hoflund, *Appl. Phys. Lett.*, 1998, **72**, 1469-1471.
4. M. Beerbom, O. Henrion, A. Klein, T. Mayer and W. Jaegermann, *Electrochim. Acta*, 2000, **45**, 4663-4672.
5. G. Hollinger, R. Skheyta-Kabbani and M. Gendry, *Physical Review B*, 1994, **49**, 11159-11167.
6. J. F. Moulder, J. Chastain and R. C. King, *Handbook of X-ray photoelectron spectroscopy: a reference book of standard spectra for identification and interpretation of XPS data*, Perkin-Elmer Eden Prairie, MN, 1992.
7. B. J. Flinn and N. S. McIntyre, *Surf. Interface Anal.*, 1990, **15**, 19-26.
8. D. Mandler and A. J. Bard, *Langmuir*, 1990, **6**, 1489-1494.
9. D. Mandler and A. J. Bard, *J. Electrochem. Soc.*, 1990, **137**, 2468-2472.
10. S. H. Kim, P. K. Mohseni, Y. Song, T. Ishihara and X. Li, *Nano Lett.*, 2014, **5**, 641-648.
11. M. DeJarld, J. C. Shin, W. Chern, D. Chanda, K. Balasundaram, J. A. Rogers and X. Li, *Nano Lett.*, 2011, **11**, 5259-5263.
